# Enhanced survival following oral and systemic *Salmonella enterica* serovar Typhimurium infection in polymeric immunoglobulin receptor knockout mice

**DOI:** 10.1371/journal.pone.0198434

**Published:** 2018-06-01

**Authors:** Kristina J. Betz, Elizabeth A. Maier, Surya Amarachintha, David Wu, Erik P. Karmele, Jeremy M. Kinder, Kris A. Steinbrecher, Monica M. McNeal, Deborah H. Luzader, Simon P. Hogan, Sean R. Moore

**Affiliations:** 1 Gastroenterology, Hepatology and Nutrition, Cincinnati Children’s Hospital Medical Center, Cincinnati, Ohio, United States of America; 2 Allergy and Immunology, Cincinnati Children’s Hospital Medical Center, Cincinnati, Ohio, United States of America; 3 Center for Autoimmune Genomics and Etiology, Cincinnati Children’s Hospital Medical Center, Cincinnati, Ohio, United States of America; 4 Infectious Disease, Cincinnati Children’s Hospital Medical Center, Cincinnati, Ohio, United States of America; 5 Division of Gastroenterology, Hepatology and Nutrition, Department of Pediatrics, University of Virginia, Charlottesville, Virginia, United States of America; New York State Department of Health, UNITED STATES

## Abstract

**Background:**

Polymeric immunoglobulin receptor (pIgR) transport of secretory immunoglobulin A (SIgA) to mucosal surfaces is thought to promote gut integrity and immunity to *Salmonella enterica* serovar Typhimurium (*S*. *Typhimurium*), an invasive pathogen in mice. To elucidate potential mechanisms, we assessed intestinal barrier function and both oral and systemic S. *Typhimurium* virulence in pIgR knockout (KO) and wildtype (WT) mice.

**Methods:**

In uninfected animals, we harvested jejunal segments for Ussing chamber analyses of transepithelial resistance (TER); mesenteric lymph nodes (mLN) for bacterial culture; and serum and stool for IgA. Separately, we infected mice either orally or intravenously (IV) with *S*. *Typhimurium* to compare colonization, tissue dynamics, and inflammation between KOs and WTs.

**Results:**

Uninfected KOs displayed decreased TER and dramatically increased serum IgA and decreased fecal IgA vs. WT; however, KO mLNs yielded fewer bacterial counts. Remarkably, WTs challenged orally with *S*. *Typhimurium* exhibited increased splenomegaly, tissue colonization, and pro-inflammatory cytokines vs. pIgR KOs, which showed increased survival following either oral or IV infection.

**Conclusions:**

Absence of pIgR compromises gut integrity but does not exacerbate bacterial translocation nor *S*. *Typhimurium* infection. These findings raise the possibility that immune adaptation to increased gut permeability and elevated serum IgA in the setting of SIgA deficiency provides compensatory protection against invasive gut pathogens.

## Introduction

IgA is the most abundant immunoglobulin class in humans and mice, and the predominant antibody found at mucosal surfaces [1]. Further, selective IgA deficiency is the most common human primary immunodeficiency [2]. The majority of IgA is produced by plasma cells in the lamina propria, where it is subsequently transported to the gut lumen by the polymeric immunoglobulin receptor (pIgR) [3]. pIgR is a glycoprotein expressed on the basolateral side of intestinal epithelial cells, where it binds to the J-chain of dimeric IgA and is transported to apical surfaces [4]. It is next cleaved by proteases, leaving part of the pIgR molecule—the secretory component—bound to the antibody to produce secretory IgA (SIgA) [5].

The mucosal surface of the gastrointestinal (GI) tract is exposed to numerous antigens and microbes, hence the GI epithelium acts as a barrier between infectious and noxious contents of the gut lumen and sterile body compartments, while allowing for the absorption of fluids, electrolytes, and nutrients [6],[7]. SIgA promotes innate immune functions, including immune exclusion, anti-inflammatory processes, and symbiosis with commensals [8,9]. Hence, SIgA is thought to play a crucial role in preserving the balance between immunity and tolerance [10,11].

Intestinal barrier (IB) dysfunction has been previously reported in pIgR knockout (KO), which lack SIgA (as well as pentameric IgM, thought to be less important) at mucosal surfaces. Evidence for IB dysfunction includes: increased *E*. *coli* specific serum IgG [12], increased bacterial growth from mesenteric lymph nodes [13], and increased serum IgA and IgG against commensal and food antigens [13]—all suggesting increased gut permeability and concomitant bacterial translocation in pIgR KO mice.

Beyond promoting gut integrity, SIgA is thought to protect against invasive gut pathogens by additional mechanisms, including: preventing pathogen-epithelial interactions[7,14]; inhibiting motility [15]; bacterial trapping within the mucus layer [16]; and retrograde transport of antigens from the basolateral epithelium to the GI lumen [17,18]. Wijburg et al. found pIgR KO mice were profoundly sensitive to low doses of oral *S*. *Typhimurium* infection vs. wildtype (WT), and pIgR KO mice transmitted *S*. *Typhimurium* more readily to other mice [19].

We conducted experiments to test the hypothesis that absence of SIgA directly impairs small IB function and resistance to *S*. *Typhimurium* invasiveness. Here, we report: 1) pIgR KO mice show decreased small intestine transepithelial resistance but fewer bacterial colonies in mesenteric lymph nodes; 2), pIgR KO mice display decreased colonization of extraintestinal tissues, milder splenomegaly, and reduced systemic inflammation relative to wild type controls following oral infection with *S*. *Typhimurium*; and 3) Surprisingly, pIgR KO mice survive significantly longer when challenged either orally or systemically with *S*. *Typhimurium*.

## Methods

### Mice

pIgR KO mice (purchased from MMRRC) on a C57BL/6 background were bred with in-house C57BL/6j mice to create heterozygotes. Heterozygotes were bred to produce homozygous dams and homozygous sires which were subsequently crossed to generate full litters of pIgR KO pups. Wild type C57BL/6j (WT) were bred in-house as controls. Uninfected mice were housed in a specific-pathogen free barrier room with a 6am-6pm light schedule. WT and KO mice were transferred to a containment mouse room with microisolator cages immediately prior to infection. To avoid pIgR KO consumption of fecal IgA and microbiota from WT controls, pIgR KO and WT mice were bred and housed separately. For euthanasia, terminal blood collection via cardiac puncture was performed under anesthesia with isoflurane followed by major organ harvest. Mice in the survival studies were monitored daily for weight, illness, and signs of obvious discomfort, distress or pain. Mice that exhibited a weight loss of greater than 20% of their starting weight were euthanized via carbon dioxide followed by cervical dislocation. There were no unexpected deaths in any of the studies. All animal procedures were conducted in accordance with Cincinnati Children’s Hospital Research Foundation Institutional Animal Care and Use Committee and were approved as part of Protocol IACUC2015-0053.

### Serum and stool IgA and IgG ELISA; stool IgA flow cytometry

Serum and stool antibody levels were quantified by ELISA as previously described [20]. To quantify total IgA coating of stool bacteria, 2–3 fecal pellets were collected into 1ml of sterile phosphate buffered saline (PBS), as previously described [21]. Pellets were homogenized by vortexing, then centrifuged at 400xg to pellet large debris. The supernatant was passed through a 70um filter and centrifuged at 8,000xg for 5 minutes. The pellet was resuspended in 1mL of 1XPBS + 5% rat serum + 5uM SYTO BC (Thermo Fisher) to stain bacterial nucleic acid and incubated for 20 minutes at 4°C. After incubation, the tube was centrifuged (8,000xg, 3 minutes). The supernatant was discarded, and the pellet washed with 1X PBS and centrifuged (8,000xg, 3 minutes). The pellet was suspended in 1mL of 1XPBS + anti-mouse IgA-PE (1:200 dilution) (Southern Biotech) or anti-mouse IgG-PI (Invitrogen catalog number 88–50400, concentrated with a Sartorius Vivaspin ultrafiltration spin column per manufacturer instructions) and incubated for 20 minutes at 4°C, then centrifuged (8,000xg, 3 minutes), and washed with 1XPBS. Finally, we centrifuged (8,000xg, 3 minutes) and resuspend in FACS Buffer. Data were collected on a BD LSR Fortessa I, and analyzed with FlowJo software.

### Ex vivo intestinal barrier function

Intestinal permeability was measured using Ussing chambers, as previously described [22]. Following sacrifice, mid-jejunal segments were excised and flushed with PBS, then opened along the mesenteric border, and mounted in Ussing diffusion chambers (exposure area of 0.30 cm^2^). Mucosal and serosal reservoirs were filled with 10ml of Krebs Ringer bicarbonate buffer. Permeability markers (FITC-dextran, 2.2 mg/ml; Sigma-Aldrich) were measured according to Forbes et al. [23]. Data for transepithelial resistance (TER) (ohm/cm^2^), short-circuit current (Isc), and flux of FITC-dextran (fmol·cm^2^·h^-1^) were normalized to the average measurements of C57BL/6 mice in each independent experiment.

### Mesenteric lymph node colony counts

Mesenteric lymph node (mLN) colony counts were performed by collection of mLNs from each mouse using sterile technique. The tissue was weighed, then homogenized in 1ml sterile PBS then 50ul was plated on TSA 5% blood agar (VWR) and incubated overnight at 37°C. Colonies were counted and colony forming units (CFU) divided by mLN weight were calculated.

### S. Typhimurium infection

*S*. Typhimurium SL1344 was grown overnight in LB broth (Invitrogen) in a shaker at 37°C. CFUs were estimated by optical density at an absorbance (A) of 600 and confirmed by growth on LB agar (VWR) with 50ug/ml streptomycin (Sigma).

Mice 8–10 weeks of age were housed 2–4 mice per cage in disposable cages and fasted in the morning for 4 hours prior to infection in the afternoon. No pre-treatment with streptomycin or sodium bicarbonate was used. Mice were gavaged with 10^9^ CFU in 150ul 100mM HEPES buffer (pH = 8.0) (Fischer Scientific) and food was replaced. Mice were followed for 2 weeks for survival.

To measure colonization, mice were sacrificed on day 7 post-infection by isoflurane and cardiac puncture. The spleen, liver, cecum, and mesenteric lymph nodes were collected and weighed for determination of bacterial load. Briefly, tissues were collected into 1ml thioglycolate (USP Alternative) using sterile technique. A 1/8” steel bead was added to each sample, and samples dissociated using a TissueLyser (Qiagen) for 3 minutes at 30 Hz for 4 cycles. Samples were diluted and plated on LB agar with 50ug/ml streptomycin. Plates were incubated for 48 hours at 37°C and counted.

For IV *Salmonella* infection, the tail vein was injected with 200ul of PBS containing 10^2−3^ CFU and mice were followed daily for survival.

### Intestinal immunohistochemistry

Histological sections from the duodenum, jejunum, ileum, and colon of pIgR KO mice and WT controls were stained for macrophages and intraepithelial lymphocytes using antibodies against F4/80 and CD 45, respectively and qualitatively compared.

Immunohistochemistry (IHC) for CD45 was performed on a robotic platform (Ventana discover Ultra Staining Module, Ventana Co., Tucson, AZ, USA). Tissue sections (4 μm) were deparaffinized using EZ Prep solution (Ventana). A heat-induced antigen retrieval protocol set for 64 min was carried out using a TRIS–ethylenediamine tetracetic acid (EDTA)–boric acid pH 8.4 buffer (Cell Conditioner 1). Endogenous peroxidases were blocked with peroxidase inhibitor (CM1) for 8 min before incubating the section with CD45 antibody (BD Pharmingen,Cat# 550286) at 1:100 dilution for 60 min at room temperature. Antigen-antibody complex was then detected using DISC. OmniMap anti-Rat HRP RUO detection system and DISCOVERY ChromoMap DAB Kit (Ventana Co.). All the slides were counterstained with hematoxylin subsequently; they were dehydrated, cleared and mounted for the assessment.

For F4/80 IHC, tissue sections were cut from each block at 4 μm thick intervals. Antigen retrieval and deparaffinization were performed in PT Link (Dako, Glostrup, Denmark) using low pH EnVision FLEX Target Retrieval Solution (Dako) for 20 min at 97°C. Immunohistochemistry was performed on a robotic platform (Autostainer, Dako). Endogenous peroxidases were blocked with peroxidase and alkaline Phosphatase blocking reagent (Dako) before incubating the sections with F4/80 antibody (AbD Serotech) at 1:200 dilution for 60 minutes at room temperature. Antigen–antibody complex was detected by using rabbit anti-rat biotin, and streptavidin HRP (Vector laboratory), and then followed by incubation with 3,3’-diaminobenzidine tetrahydrochloride (DAB+) chromogen (Dako). All the slides were counterstained with hematoxylin subsequently; they were dehydrated, cleared and mounted for the assessment.

### Serum cytokines

Serum cytokine concentrations were determined by enzyme-linked immunosorbent assay (ELISA) using Milliplex^TM^ Multiplex kits (Millipore) according to manufacturer’s protocol. Briefly, in a 96 well black plate, 25μL sample in duplicate was incubated with 25μL antibody coated beads overnight at 4°C while shaking. Plates were washed twice and 25μL of secondary antibody was added and incubated at room temperature for 1 hour while shaking. Finally, 25μL of streptavidin-RPE was added to the secondary antibody and incubated for 30 minutes at room temperature with shaking. Plates were washed twice and 100μL of sheath fluid was added. Plates were shaken for 5 minutes and read using Luminex technology on the Bio-Plex^TM^ (Bio-Rad). Concentrations were calculated from standard curves using recombinant proteins and reported in pg/ml.

### Statistical methods

All statistical analyses were performed using GraphPad Prism, version 5. F-tests were applied to establish Gaussian distribution of samples. A two-sample t test or ANOVA was applied when groups displayed equal variances. Where appropriate, log transformation was applied to normalize antibody levels. The Mann-Whitney or Kruskal-Wallis tests were applied to non-parametric data. Log-rank test was used to compare survival curves. *P* values less than 0.05 were considered significant. Error bars on graphs and data represent mean ± SEM.

## Results

### Increased serum IgA and decreased stool IgA and IgA-coated bacteria in pIgR KO mice

**Fig 1:**
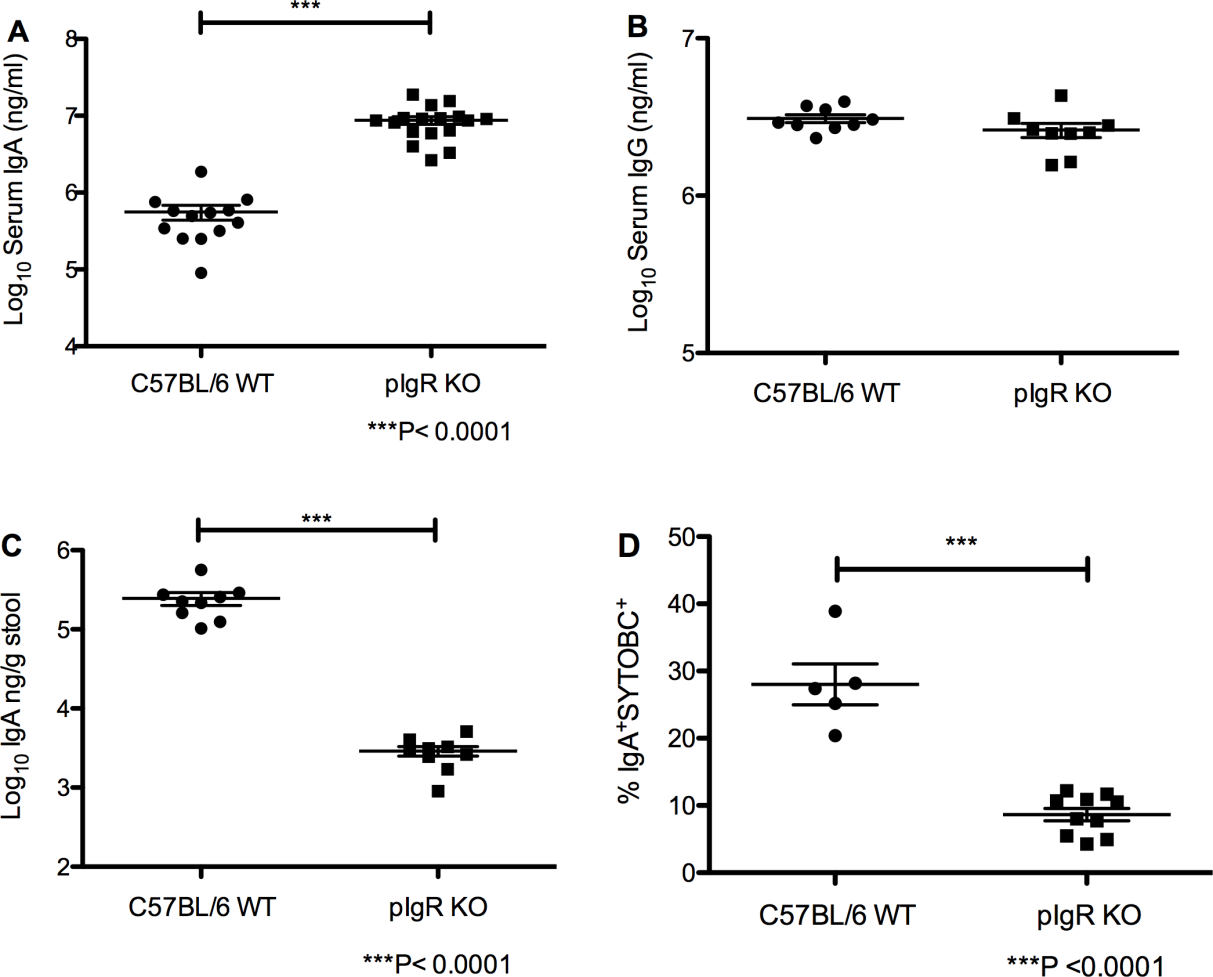
Elevated serum IgA, decreased stool IgA, and decreased IgA coating of fecal bacteria in pIgR KO mice. (A) Serum IgA is significantly increased in pIgR KO mice compared to C57BL/6 mice (*P*<0.0001, Mann-Whitney). (B) No significant difference in the serum IgG between pIgR KO and C57BL/6 mice. (C) Stool IgA is significantly decreased in pIgR KO mice compared to C57BL/6 mice (*P*<0.0001, Mann-Whitney). (D) pIgR KO mice showed a 3-fold reduction in the percentage of stool bacteria coated with IgA (*P*<0.0001, Mann-Whitney).

Serum IgA and IgG antibodies were first measured in 8–10 week old C57BL/6 and pIgR KO mice to verify expected changes based on pIgR loss. As expected, serum IgA was elevated in knockout mice compared to controlC57BL/6 mice (8.7x10^6^±1.0x10^6^ and 5.6x10^5^±1.2x10^5^ ng/ml in pIgR and C57BL/6 mice respectively, *P*<0.0001, 2-sample T-test) (Fig 1A). However, no differences were found in serum IgG in KO mice compared to C57BL/6 mice (2.6x10^6^±2.7x10^5^ vs. 3.1x10^6^±1.8x10^5^ ng/ml, respectively, Mann-Whitney test) (Fig 1B).

As expected, stool IgA is significantly reduced in knockout mice compared to control mice (2.9x10^3^±4.1x10^2^ vs. 2.5x10^5^±4.5 x10^4^ ng/g of stool, respectively, *P*<0.0001, Mann-Whitney) (Fig 1C). In addition, KO mice showed significant reductions in the percentage of stool bacteria coated with IgA (8.6±0.9% for pIgR KO mice and 28.0±3.0% for C57BL/6 mice, *P*<0.0001, Mann-Whitney) (Fig 1D, representative plots S1 Fig). Stool IgG is significantly increased in knockout mice compared to control mice (2.1±0.12 for pIgR KO mice and 1.6±0.0057 for C57BL/6 mice) (S2 Fig).

### *Ex vivo* intestinal barrier defects in pIgR KO mice

**Fig 2:**
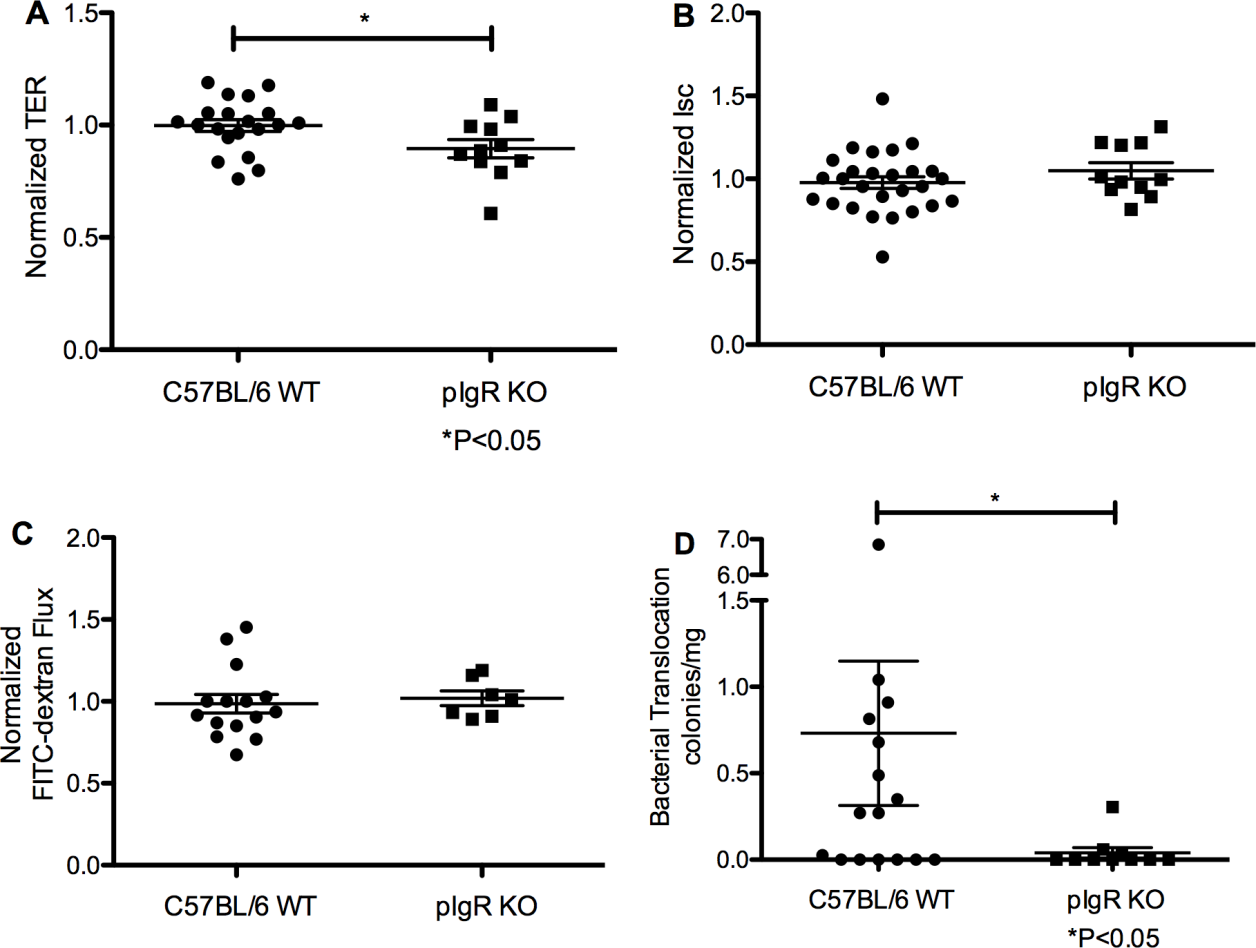
Small intestinal transepithelial resistance is impaired, but translocation of bacteria is diminished, in pIgR KO mice. Ussing chamber studies of mid-jejunal sections revealed a modest decrease in (A) transepithelial resistance in pIgR knockout mice as compared to C57BL/6 mice (*P*<0.05, by t-test), and no difference in measures of (B) short circuit current, or (C) FITC-dextran flux. Data are representative of 3 experiments. (D) Fewer aerobic bacterial colonies were recovered from homogenized mesenteric lymph nodes of pIgR KO mice compared to C57BL/6 mice (*P*<0.05, Mann-Whitney). Data are representative of 6 experiments.

Intestinal barrier function (IB) was measured *ex vivo* in 8–10 week old mice using mid-jejunal segments mounted in Ussing chambers to characterize the IB defect in the pIgR KO mice. Electrophysiological and permeability measurements revealed normalized TER was lower in pIgR KO mice vs C57BL/6 mice (0.90±0.04 vs.1.00±0.03, respectively, *P*<0.05, 2-sample t-test) (Fig 2A). Additionally, normalized Isc, a measure of intestinal ion transport, was similar between pIgR KO mice and C57BL/6 mice (1.05±0.05 vs. 0.98±0.04, respectively, *P* = 0.29, 2-sample t-test) (Fig 2B). Finally, we detected no differences in normalized FITC-dextran flux across the intestinal tissues between pIgR KO mice and C57BL/6 mice (1.02±0.05 vs. 0.99±0.06, respectively, *P* = 0.71, 2-sample t-test) (Fig 2C). Non-normalized means are shown in S1 Table. These data indicate small intestinal barrier function is only mildly impaired in pIgR KO mice. IB function was also evaluated using colonic segments, with no differences in TER, Isc, or FITC-dextran flux detected (S3 Fig).

### Decreased blood bacterial translocation in pIgR KO mice

Non-specific bacterial counts were quantified from the mesenteric lymph nodes of pIgR KO mice and control mice as an indirect measure of intestinal permeability and bacterial translocation. We recovered significantly fewer aerobic colonies from the mesenteric lymph nodes of pIgR KO mice compared to C57BL/6 mice (0.04±0.03 vs. 0.74±0.42 CFU/g, respectively, *P*<0.05, Mann-Whitney) (Fig 2D). We detected no significant group differences in lymph node weights (S4 Fig).

### Increased survival of pIgR KO mice following oral *S*. *Typhimurium* challenge

**Fig 3:**
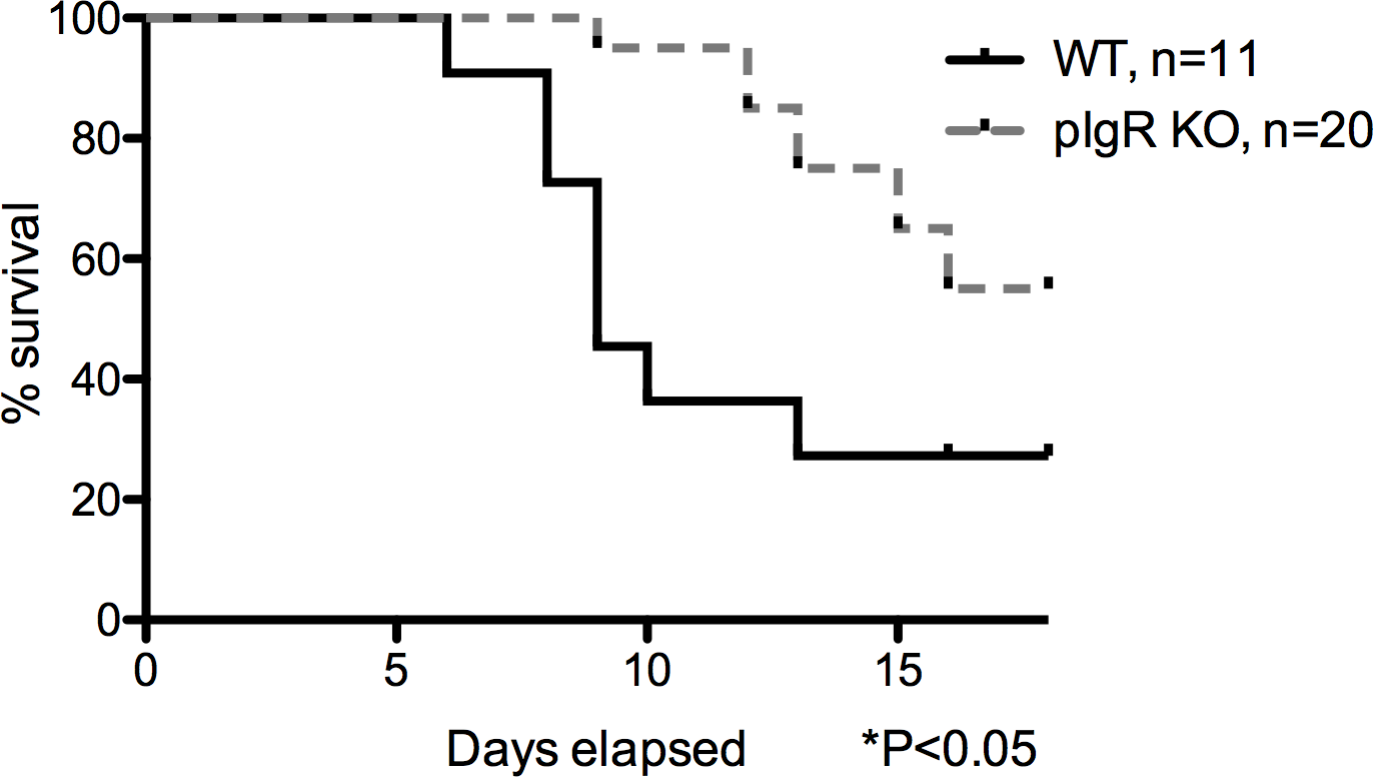
Increased survival of pIgR KO following high-dose oral *S*. *Typhimurium* challenge. Mice were gavaged with 10^9^ CFU of *S*. *Typhimurium* following a 4-hour fast and followed for 2 weeks. Mean survival for C57BL/6 mice was statistically significantly shorter than the pIgR KO mice (*P*<0.05, log-rank Mantel-Cox test). Data are representative of 3 experiments, with equivalent infectious doses between KO and WT in each experiment.

To test the susceptibility to infection of pIgR KO mice following an oral *S*. *Typhimurium* challenge, we fasted mice for 4 hours then orally gavaged 10^9^ CFU of *S*. *Typhimurium*. The pIgR KO mice showed an increased median survival (*P*<0.05 by the log-rank Mantel-Cox test) as compared to the C57BL/6 mice (undefined for pIgR KO mice and 9 days for C57BL/6 mice, Fig 3), with 55% of pIgR KO mice and 27% of C57BL/6 mice alive at 16 days post infection.

### Protection from systemic colonization by *S*. *Typhimurium* in pIgR KO mice

**Fig 4:**
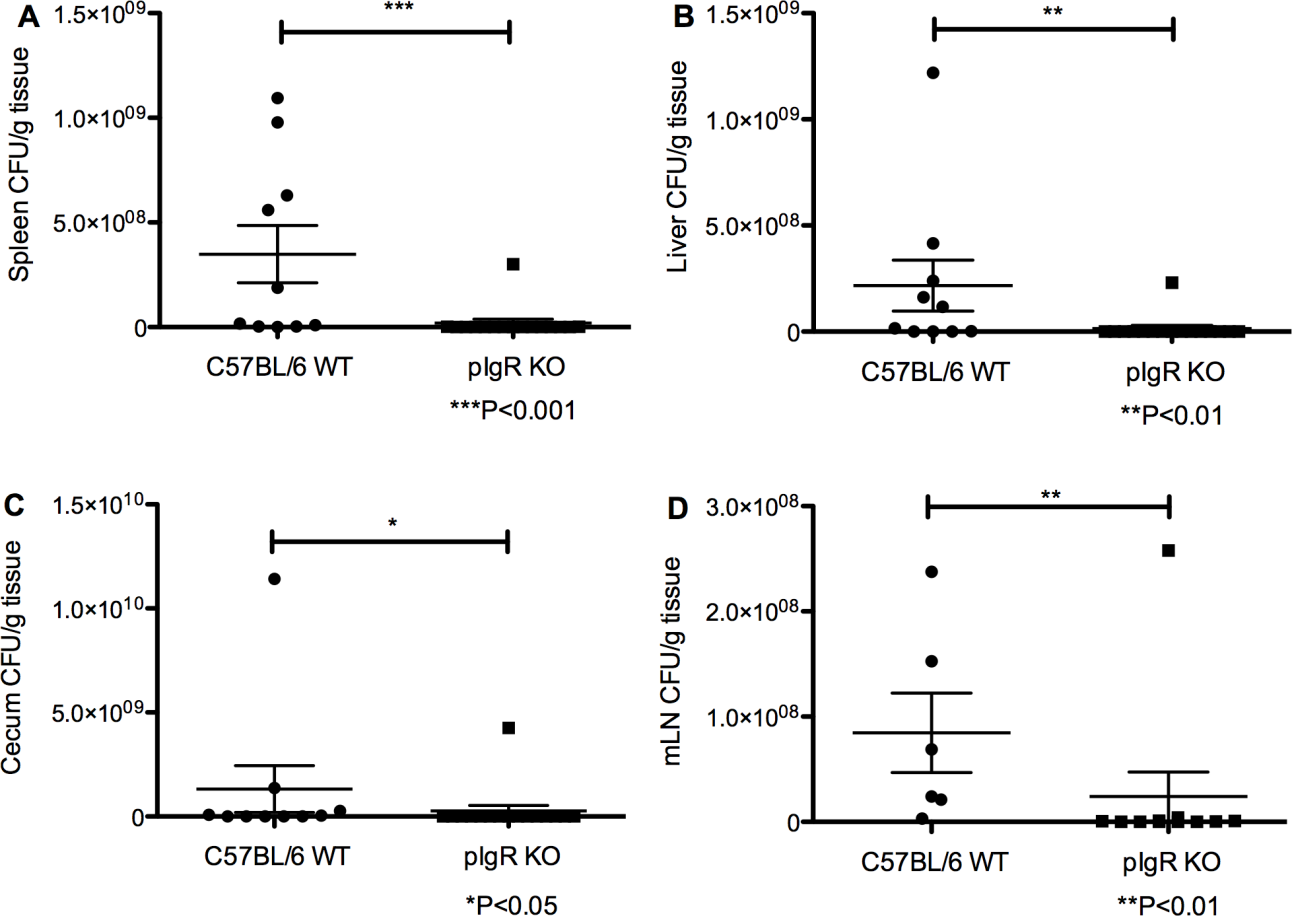
Systemic and intestinal colonization with *S*. *Typhimurium* is diminished in pIgR KO mice following oral challenge. *S*. *Typhimurium* CFUs were significantly higher in C57BL/7 mice compared to pIgR KO mice on day 7 post-oral infection in the (A) spleen (*P*<0.001, Mann-Whitney test), (B) liver (*P*<0.01, Mann-Whitney), (C) cecum (*P*<0.05, Mann-Whitney test), and (D) mLN (*P*<0.01, Mann-Whitney). Data are representative of 3 experiments.

To assess the systemic spread of *S*. *Typhimurium* in pIgR KO compared to C57BL/6, we orally gavaged mice with *S*. *Typhimurium* as described above, and sacrificed mice at 7 days post-infection to collect tissues for evaluation of bacterial growth. C57BL/6 mice had significantly increased growth based on CFUs from spleen (1.9x10^7^±1.9x10^7^ CFU/g pIgR KO and 3.5x10^8^±1.4x10^8^ CFU/g for C57BL/6, *P*<0.001 by the Mann-Whitney test) (Fig 4A), liver (1.5x10^7^±1.4x10^7^ CFU/g pIgR KO and 2.2x10^8^±1.2x10^8^ CFU/g for C57BL/6, *P*<0.01 by the Mann-Whitney test) (Fig 4B), cecum (2.7x10^8^±2.7x10^8^ CFU/g pIgR KO and 1.3x10^9^±1.1x10^9^ CFU/g for C57BL/6, *P*<0.05 by the Mann-Whitney test) (Fig 4C), and mLNs (2.4x10^7^±2.3x10^7^ CFU/g pIgR KO and 8.5x10^7^±3.8x10^7^ CFU/g for C57BL/6, *P*<0.01 by the Mann-Whitney test) compared to pIgR KO mice (Fig 4D).

**Fig 5:**
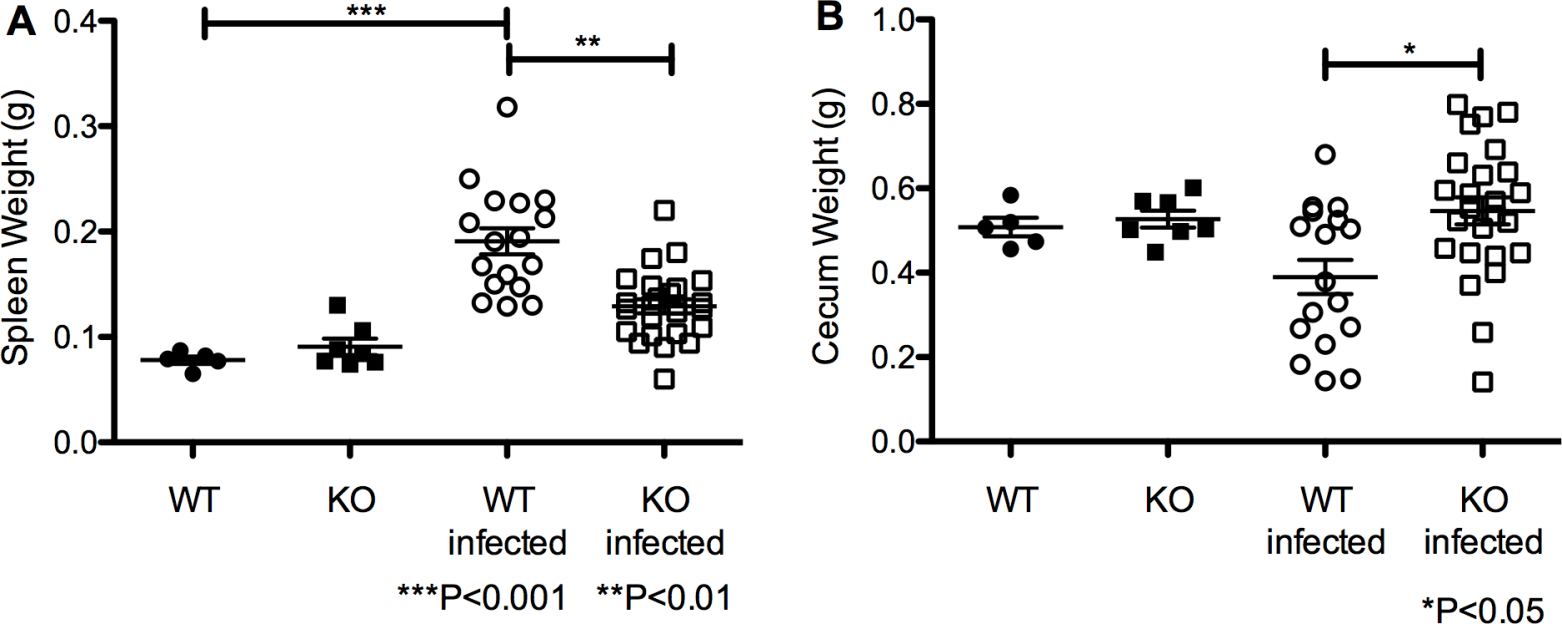
Intestinal and systemic indicators of *S*. *Typhimurium* infection intensity are reduced in pIgR KO mice following oral challenge. (A) Spleen weight was significantly elevated in C57BL/6 mice 7 days post-oral infection compare to both uninfected C57BL/6 (*P*<0.001) and infected pIgR KO (*P*<0.01, Kruskal-Wallis with Dunn multiple comparison test). (B) Cecum weight was significantly diminished in C57BL/6 mice compared to pIgR KO mice at 7 days post-oral infection (*P*<0.05, Kruskal-Wallis with Dunn multiple comparison test). Data are representative of 3 experiments. Baseline weights of spleen and cecum did not differ in uninfected C57BL/6 and pIgR KO mice.

Similarly, pIgR KO mice were found to be protected from splenomegaly and reduction in cecum weight, two known sequelae of *S*. *Typhimurium* infection [24,25], relative to C57BL/6. Organ weight was compared between WT and KO uninfected mice, uninfected to infected for both WT and KO mice, and WT and KO infected groups. No differences were detected in spleen or cecum weight at baseline between the two groups (Fig 5A and 5B). Spleen weights in infected pIgR KO mice vs WT mice were significantly lower (0.13±0.0`g vs. 0.19±0.01g, respectively, *P*<0.01, ANOVA with Dunn multiple comparisons test) (Fig 5A), while cecum weights were significantly higher in pIgR KO vs WT mice (0.55±0.03g vs. 0.39±0.48g, respectively, *P*<0.05, ANOVA with Dunn multiple comparisons test) (Fig 5B).

### Diminished serum cytokines in pIgR KO mice following oral *S*. *Typhimurium* challenge

**Fig 6:**
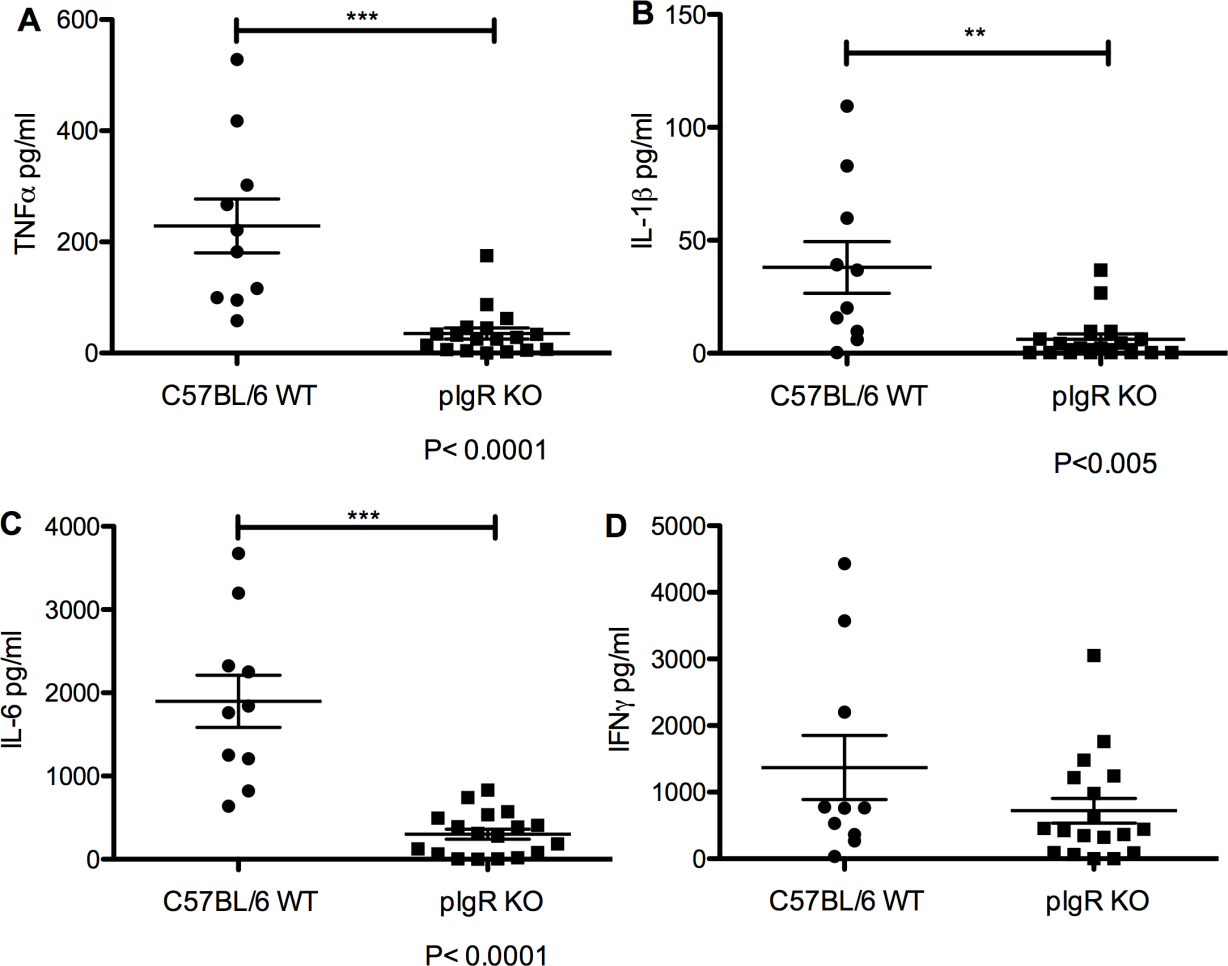
Serum pro-inflammatory cytokines TFNα, IL-1β, and IL-6 are significantly lower in pIgR KO mice vs wildtype mice 7 days post oral *S*. *Typhimurium* infection. C57BL/6 mice showed significantly increased (A) TNFα (*P*<0.0001, Mann-Whitney), (B) IL-1β (*P*<0.005, Mann-Whitney test), and (C) IL-6 (*P*<0.0001, Mann-Whitney) compared to pIgR KO. (D) No difference in IFNγ levels between groups.

To assess the systemic inflammatory reaction to *S*. *Typhimurium* spread in pIgR KO mice compared to wildtype mice, serum was collected for cytokine analysis by Luminex arrays 7 days post-oral infection. C57BL/6 mice showed significantly increased TNFα (35.3±9.9 pg/ml for pIgR KO and 228.9±48.4pg/ml for C57BL/6, *P*<0.0001, Mann-Whitney) (Fig 6A), IL-1β (6.2±2.4pg/ml for pIgR KO and 38.0±11.4 pg/ml for C57BL/6, *P*<0.005, Mann-Whitney) (Fig 6B), and IL-6 (301.7±61.5 pg/ml for pIgR KO and 1898.0±313.0 pg/ml for C57BL/6, *P*<0.0001, Mann-Whitney) (Fig 6C) as compared to pIgR KO. We detected no difference in IFNγ levels between groups (Fig 6D). In addition, serum levels of anti-inflammatory cytokine IL-10 were significantly decreased in pIgR KO mice relative to controls (S5 Fig). Qualitatively, we detected no significant differences in the amount or distribution of either macrophages (F4/80 antibody staining, S6 Fig) or intraepithelial lymphocytes (CD45 antibody staining, S7 Fig) in the duodenum, jejunum, ileum, or colon of pIgR KO mice as compared to controls.

### Increased survival following intravenous *S*. *Typhimurium* challenge

**Fig 7:**
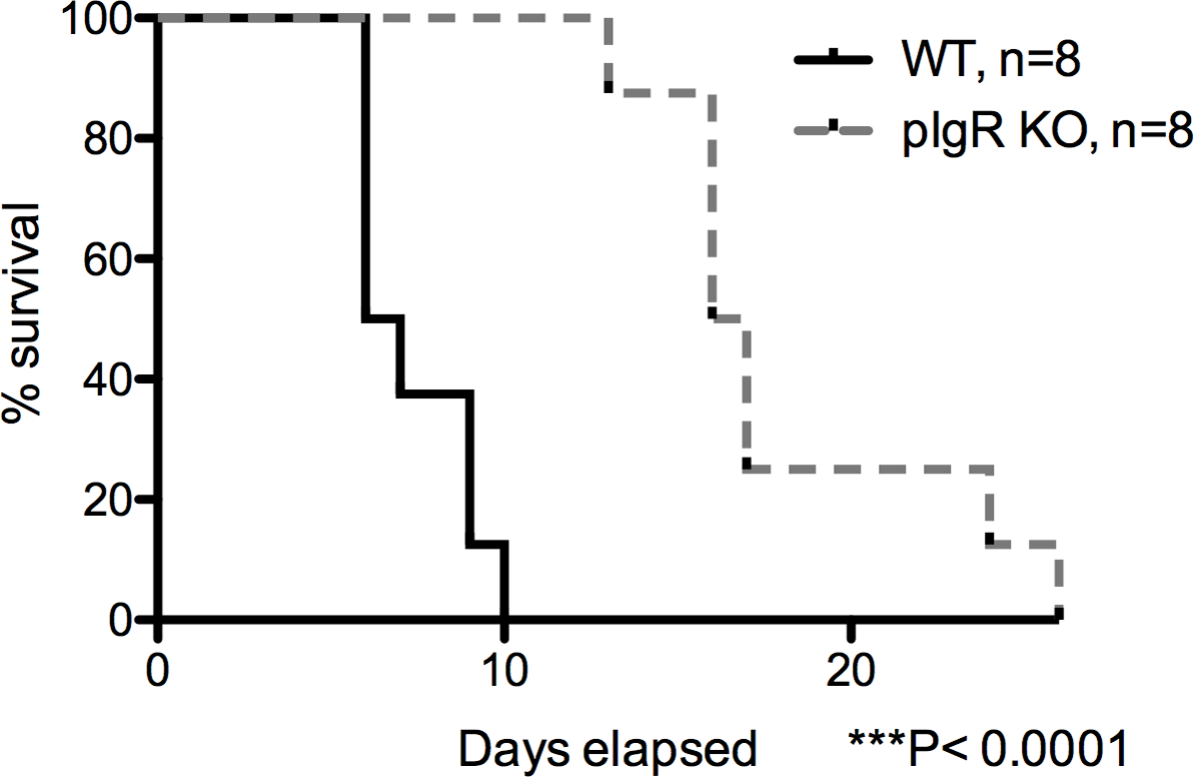
Increased survival of pIgR KO following intravenous *S*. *Typhimurium* challenge. Mice were injected IV via the tail vein with 3.2x10^2^ CFU of *S*. *Typhimurium* and followed for 26 days. Mean survival for C57BL/6 mice was statistically significantly shorter than the pIgR KO mice (*P*<0.0001, log-rank Mantel-Cox test). Data are representative of 2 experiments.

The findings of: 1) a baseline gut barrier defect and 2) decreased systemic inflammation, with enhanced survival following oral *S*. *Typhimurium* infection in pIgR KO mice, prompted us to hypothesize that pIgR KO mice develop compensatory immune mechanisms that confer protection to systemic *S*. *Typhimurium* infections. To test this hypothesis, pIgR KO were challenged with 3.2x10^2^ CFU of *S*. *Typhimurium* via tail vein injection. KO mice exhibited dramatically prolonged survival compared to WT mice (16.5 days pIgR KO and 6.5 days for C57BL/6, *P*<0.0001 by the log-rank Mantel-Cox test) (Fig 7).

## Discussion

In this report, we examined intestinal barrier (IB) function and susceptibility to infection of pIgR knockout mice based on the hypothesis that pIgR plays an important role in gut integrity and protection against invasive gut pathogens. Indeed, we found that mice lacking pIgR have a modest decrease in jejunal transepithelial resistance; however, we saw no concomitant increase in FITC-dextran permeability and recovered fewer commensal bacteria from pIgR KO mesenteric lymph nodes, indicating both intact paracellular permeability and enhanced clearance of translocated commensals. Further, we found pIgR KO mice were less sensitive to high-dose oral *S*. *Typhimurium* infection, as well as low-dose intravenous *S*. *Typhimurium* infection. Thus, our data suggest that although pIgR is a key molecule for SIgA transport but perhaps less critical than previously thought for IB function or protection against *S*. *Typhimurium* infection.

Based on previous studies, we expected to find increased serum IgA and IgG, and decreased stool IgA in pIgR KO mice [26,27]. We did confirm a significant increase in serum IgA and a decrease in stool IgA, however we did not find increased serum IgG in pIgR KO mice. Previous investigators suggested increased serum IgG was due to increased systemic contact with gut bacteria, as indicated by increased serum IgG against commensals [27]. In contrast, the pIgR KO mice in our colony showed decreased bacteria in their mesenteric lymph nodes, which may explain this discrepancy, as well as our findings that pIgR KO mice exhibited increased fecal IgG levels vs wildtype controls.

We detected an 85-fold decrease in total stool IgA in pIgR KO compared to WT, yet only a 3.5-fold drop in IgA-coated stool bacteria. Two potential explanations for this finding include: 1) leakage of serum IgA to the gut lumen across a damaged gut barrier, with subsequent coating of gut bacteria and 2) a shift in the gut microbiome of these mice, as has been previously shown in pIgR KOs [28], and subsequent selection for inflammation-driving bacteria targeted by IgA [29].

Several studies of pIgR KO mice imply a gut integrity defect based on secondary measures, including increased serum *E*.*coli*-specific IgG [12], increased serum IgA and IgG against commensal and food antigens [13], and increased bacterial growth from mLNs [13,28]. Our present studies complement these earlier findings by direct measurements of altered intestinal physiology in pIgR KO mice using Ussing chambers. Using this technique, we detected modest decreases in jejunal TER in pIgR KOs. However, we found no differences in Isc or, interestingly, FITC-dextran flux. These data suggest jejunal “leakiness” is increased in the absence of pIgR [30], yet the epithelium is still able to act as an effective barrier to larger molecules like FITC-dextran. One limitation of our study is we did not assess other regions of the small intestine, which may have revealed more significant differences.

We found pIgR KOs in our facility yielded significantly fewer bacteria from mLNs. It is plausible that increased serum and gut lamina propria IgA in pIgR KOs could play a role in quickly clearing translocated bacteria to suppress mLN colonization. This would also be consistent with the results from our infection studies. The increase in the stool IgG of the pIgR KO mice may also be serving as a protective compensatory mechanism to prevent bacterial translocation. Kato-Nagaoka et al. recently confirmed earlier reports of increased bacterial counts from the mLNs of pIgR KO mice, however they detected lower values than previous studies [13,28,31]. Several factors, including technical details of the assay and variability in local animal facility microbiomes may account for these differences. Additionally, our study used mice 8–10 weeks of age, which may be too young to detect differences in IB. As reported by Kato-Nagaoka et al., CD8αβ+αβ-IELs accumulate in the pIgR KOs intestine with aging and contribute to IB break-down [31].

Wijburg et al. reported pIgR KOs are profoundly sensitive to low-dose *S*. *Typhimurium* challenge compared to C57BL/6 mice [19]. We tested similar doses WT and KO mice but found no differences between KO and WT (data not shown), so proceeded with a high dose challenge. We were surprised to find pIgR KOs were partially resistant to *S*. *Typhimurium* lethality, when administered at high doses either orally and intravenously. There are several potential explanations for these differences. First, pIgR KOs in the present study showed less baseline bacterial translocation compared to previous reports. Second, intestinal microbiota are critical in mediating the clearance of *Salmonella* from the intestine, hence local differences in microbiota might account for different outcomes following infection [32]. Last, we used the equivalent strain of *S*. *Typhimurium* reported by Wijburg et al., however the possibility of subsequent strain mutations may have influenced our results.

In the present study, pIgR KO mice showed decreased bacterial counts in the liver and spleen following oral infection, suggesting a decreased number of bacteria seeding these tissues [33,34]. Previous reports demonstrate B-cell deficient mice are more susceptible to *S*. *Typhimurium* infection and indicate an antibody-independent function of B-cells is protective against infection [35]. pIgR KO mice have both increased B-cell counts and increased serum IgA [27]. Supporting Figs 6 and 7 provide preliminary evidence that the number and distribution of macrophages and lymphocytes are not appreciably different between the pIgR KO mice and wildtypes. While not addressed in this paper, differences in M cell number or epithelial cell turnover could also play a role in the immune differences of the mice. Hence, the increase of B-cell counts, serum IgA, and stool IgG might have played a role in enhancing the survival of pIgR KO mice following either oral or systemic *S*. *Typhimurium* infection.

We found wildtype mice had significantly elevated serum pro-inflammatory cytokines following oral infection. The interaction between *S*. *Typhimurium* pathogen-associated molecular patterns with host receptors leads to the recruitment of macrophages and neutrophils that produce TNFα, IL-1β, IL-6, and IFNγ [36]. We found three of these four cytokines were significantly elevated in the serum of infected wildtype mice compared to pIgR KOs. These data are consistent with our findings of increased bacterial counts in peripheral organs, as increased bacterial presence would initiate increased recruitment of phagocytes. Inflammatory monocytes and neutrophils are also known to produce inducible nitric oxide synthase [37]. While these molecules are important for the killing of *S*. *Typhimurium*, uncontrolled activation of the innate immune system leads to aberrant coagulation, vasodilation leading to hypotension, tissue injury, and finally death. Given the high levels of serum cytokines detected, it is likely these mechanisms played a role in the early death of the wildtype mice.

Although we detected significant differences in our infection model, we were limited by studying only high-dose *S*. *Typhimurium* challenges, without the use of sodium bicarbonate pre-treatment, which might have exerted different effects vs. low-dose challenges with pre-treatment. We did not characterize the microbiome of pIgR KOs and C57BL/6 mice housed in our facility, which may have influenced our results. Finally, to better understand the temporal dynamics of *S*. *Typhimurium* infection in the setting of SIgA deficiency, it would be helpful in future studies to sacrifice mice at earlier time-points following oral infection to quantify bacterial invasion into Peyer’s patches and mLNs in the first few hours of the infection.

The literature supports a critical role for non-functional NRAMP1 in Salmonella susceptibility in C57BL/6 mice [38,39]. However, we identified no articles examining a dual role for NRAMP1 and pIgR in pathogen susceptibility. We sequenced NRAMP1 in several mice from our pIgR KO colony and all tested were heterozygous for non-functional NRAMP1, indicating the functional allele was present (albeit randomly) in our colony, despite 5 backcrosses to C57BL/6 mice at MMRC and additional crosses to our in-house C57BL/6 mice. Because we do not know the NRAMP1 status of each animal studied, it is impossible for us to fully ascertain the degree to which NRAMP1 status modulated the differences we observed between pIgR KO and WT mice. Nonetheless, we detected remarkably homogeneous effects of pIgR deletion on key outcomes: tissue injury (Fig 5) and systemic inflammation (Fig 6), suggesting the absence of pIgR exerted a far stronger protective influence against infection than would be expected if randomly present, functional NRAMP1 were the primary determinant of the observed outcomes we observed. However, it is possible the random presence of functional NRAMP1 may have partially accounted for the wider variability in survival (Figs 3 and 7) and systemic and intestinal colonization with *S*. *Typhimurium* in pIgR KO mice vs WT mice (Fig 4). Thus, despite a lack of comprehensive data on the potential role of NRAMP1, we feel our findings represent an advance by revealing the surprising finding of enhanced resistance to invasive bacteria in pIgR KO mice with compromised intestinal barrier function.

More broadly, our results have implications for understanding why selective IgA deficiency is a common primary human immunodeficiency with diverse clinical manifestations. Selective IgA deficiency is associated with an increased risk for common infections and autoimmune disorders, but only rarely linked to life-threatening bloodstream infections [2]. If our finding that SIgA deficiency in young mice confers partial protection against invasive gut pathogens is applicable to human hosts, selective IgA deficiency may therefore represent a balanced polymorphism, enhancing survival against deadly infections early in life, but predisposing to common infections and autoimmunity in older individuals. In conclusion, we have demonstrated that pIgR promotes IB function but is not critical for protection against *S*. *Typhimurium* infection. Redundancies in innate immunity or adaptation to gut leakiness in the context of SIgA deficiency may compensate for these losses while maintaining homeostasis and the ability to fight infection.

## Supporting information

S1 Fig Representative dot plots of IgA bacterial staining in stool. (A) Isotype control staining of C57BL6 stool. (B) Representative plot of IgA staining of bacteria in stool of C57BL6 mouse. (C) Representative plot of IgA staining of bacteria in stool of pIgR KO mouse.
(TIFF)

S2 Fig Elevated stool IgG in pIgR KO mice. Stool IgG is significantly increased in knockout mice compared to control mice (P<0.0002, Mann-Whitney). Data from eight pIgR KO mice and age-matched WT controls.
(TIFF)

S3 Fig No differences in ex vivo measures of colonic ion transport or permeability between WT and KO mice. Ussing chamber studies of colonic sections revealed no differences in (A) transepithelial resistance, (B) short circuit current, or (C) FITC-dextran flux.
(TIFF)

S4 Fig Mesenteric lymph node weight from mice on day 7 following high-dose oral *S*. *Typhimurium* challenge. No difference was detected in the weight of mesenteric lymph nodes of pIgR KO mice compared to WT mice.
(TIFF)

S5 Fig Serum anti-inflammatory cytokine IL-10 is significantly lower in pIgR KO mice vs wildtype mice 7 days post oral *S*. *Typhimurium* infection. (TIFF)

S6 Fig Similar amount and distribution of CD45 (lymphocyte marker) stained cells in the intestines of pIgR KO and wildtype mice. (A) duodenum, (B) jejunum, (C) ileum, (D) colon of wildtype mice and (E) duodenum, (F) jejunum, (G) ileum, (H) colon of pIgR KO mice. Representative images chosen from eight pIgR KO mice and age-matched WT controls.
(TIFF)

S7 Fig Similar amount and distribution of F4/80 (macrophage marker) stained cells in the intestines of pIgR KO and wildtype mice. (A) duodenum, (B) jejunum, (C) ileum, (D) colon of wildtype mice and (E) duodenum, (F) jejunum, (G) ileum, (H) colon of pIgR KO mice. Representative images chosen from eight pIgR KO mice and age-matched WT controls.
(TIFF)

S1 Table Non-normalized means of Ussing chamber studies of mid-jejunal sections from Fig 2. (TIFF)

## References

[1] Macpherson A, McCoy K, Johansen F-E, Brandtzaeg P. The immune geography of IgA induction and function. Mucosal Immunol. 2008;1(1):11–22. doi: 10.1038/mi.2007.6 19079156 10.1038/mi.2007.6

[2] Yel L. Selective IgA deficiency. J Clin Immunol. 2010 1;30(1):10–6. doi: 10.1007/s10875-009-9357-x 20101521 10.1007/s10875-009-9357-x PMC2821513

[3] Pabst O. New concepts in the generation and functions of IgA. Nat Rev Immunol. 2012;12(12):821–32. doi: 10.1038/nri3322 23103985 10.1038/nri3322

[4] Asano M, Komiyama K. Polymeric immunoglobulin receptor. J Oral Sci. 2011 6;53(2):147–56. 21712618 10.2334/josnusd.53.147

[5] Mostov KE, Deitcher DL. Polymeric immunoglobulin MDCK cells transcytoses receptor IgA expressed in MDCK cells transcytoses IgA. Cell. 1986;46:613–21. 3524859 10.1016/0092-8674(86)90887-1

[6] Tilg H, Moschen AR. Food, Immunity, and the Microbiome. Gastroenterology. 2015;148(6):1107–19. doi: 10.1053/j.gastro.2014.12.036 25575570 10.1053/j.gastro.2014.12.036

[7] Corthesy B, Kraehenbuhl J. Antibody-mediated protection of mucosal surfaces. Curr Top Microbiol Immun. 1999;236:93–111. 10.1007/978-3-642-59951-4_6 9893357

[8] Corthésy B. Multi-faceted functions of secretory IgA at mucosal surfaces. Front Immunol. 2013;4(July):185. 23874333 10.3389/fimmu.2013.00185 PMC3709412

[9] Mestecky J, Russell MW, Elson CO. Intestinal IgA: novel views on its function in the defence of the largest mucosal surface. Gut. 1999;44(1):2–5. 9862815 10.1136/gut.44.1.2 PMC1760065

[10] Corthésy B. Role of secretory IgA in infection and maintenance of homeostasis. Autoimmun Rev. 2013;12(6):661–5. doi: 10.1016/j.autrev.2012.10.012 23201924 10.1016/j.autrev.2012.10.012

[11] Peterson D a McNulty NP, Guruge JL Gordon JI. IgA response to symbiotic bacteria as a mediator of gut homeostasis. Cell Host Microbe. 2007;2(5):328–39. doi: 10.1016/j.chom.2007.09.013 18005754 10.1016/j.chom.2007.09.013

[12] Johansen FE, Pekna M, Norderhaug IN, Haneberg B, Hietala MA, Krajci P, et al Absence of epithelial immunoglobulin A transport, with increased mucosal leakiness, in polymeric immunoglobulin receptor/secretory component-deficient mice. J Exp Med. 1999;190(7):915–22. 10510081 10.1084/jem.190.7.915 PMC2195652

[13] Sait LC, Galic M, Price JD, Simpfendorfer KR, Diavatopoulos D a, Uren TK, et al Secretory antibodies reduce systemic antibody responses against the gastrointestinal commensal flora. Int Immunol. 2007;19(3):257–65. doi: 10.1093/intimm/dxl142 17255112 10.1093/intimm/dxl142

[14] Childers NK, Bruce MG, McGhee JR. Molecular mechanisms of immunoglobulin A defense. Annu Rev Microbiol. 1989;43:503–36. doi: 10.1146/annurev.mi.43.100189.002443 2508540 10.1146/annurev.mi.43.100189.002443

[15] Forbes SJ, Eschmann M, Mantis NJ. Inhibition of Salmonella enterica serovar Typhimurium motility and entry into epithelial cells by a protective antilipopolysaccharide monoclonal immunoglobulin A antibody. Infect Immun. 2008;76(9):4137–44. doi: 10.1128/IAI.00416-08 18625740 10.1128/IAI.00416-08 PMC2519396

[16] Rogier EW, Frantz AL, Bruno MEC, Kaetzel CS. Secretory IgA is Concentrated in the Outer Layer of Colonic Mucus along with Gut Bacteria. Pathog (Basel, Switzerland). 2014;3(2):390–403. 10.3390/pathogens3020390 PMC4243452 25437806

[17] Kaetzel CS, Robinson JK, Chintalacharuvu KR, Vaerman JP, Lamm ME. The polymeric immunoglobulin receptor (secretory component) mediates transport of immune complexes across epithelial cells: a local defense function for IgA. Proc Natl Acad Sci U S A. 1991;88(19):8796–800. 1924341 10.1073/pnas.88.19.8796 PMC52597

[18] Robinson JK, Blanchard TG, Levine a D, Emancipator SN, Lamm ME. A mucosal IgA-mediated excretory immune system in vivo. J Immunol. 2001;166(6):3688–92. 11238608 10.4049/jimmunol.166.6.3688

[19] Wijburg OLC, Uren TK, Simpfendorfer K, Johansen F-E, Brandtzaeg P, Strugnell R a. Innate secretory antibodies protect against natural Salmonella Typhimurium infection. J Exp Med. 2006;203(1):21–6. doi: 10.1084/jem.20052093 16390940 10.1084/jem.20052093 PMC2118088

[20] Ward RL, Bernstein DI, Young EC, Sherwood JR, Knowlton DR, Schiff GM. Human rotavirus studies in volunteers: determination of infectious dose and serological response to infection. J Infect Dis. 1986;154(5):871–80. 3021869 10.1093/infdis/154.5.871

[21] Bunker JJ, Flynn TM, Koval JC, Shaw DG, Meisel M, McDonald BD, et al Innate and Adaptive Humoral Responses Coat Distinct Commensal Bacteria with Immunoglobulin A. Immunity. 2015;43(3):541–53. doi: 10.1016/j.immuni.2015.08.007 26320660 10.1016/j.immuni.2015.08.007 PMC4575282

[22] Ueno PM, Oriá RB, Maier E a, Guedes M, de Azevedo OG, Wu D, et al Alanyl-glutamine promotes intestinal epithelial cell homeostasis in vitro and in a murine model of weanling undernutrition. Am J Physiol Gastrointest Liver Physiol. 2011 10;301(4):G612–22. doi: 10.1152/ajpgi.00531.2010 21799183 10.1152/ajpgi.00531.2010 PMC3191556

[23] Forbes EE, Groschwitz K, Abonia JP, Brandt EB, Cohen E, Blanchard C, et al IL-9- and mast cell-mediated intestinal permeability predisposes to oral antigen hypersensitivity. J Exp Med. 2008;205(4):897–913. doi: 10.1084/jem.20071046 18378796 10.1084/jem.20071046 PMC2292227

[24] Barthel M, Hapfelmeier S, Quintanilla-martínez L, Kremer M, Rohde M, Hogardt M, et al Pretreatment of Mice with Streptomycin Provides a Salmonella enterica Serovar Typhimurium Colitis Model That Allows Analysis of Both Pathogen and Host. 2003;71(5):2839–58. doi: 10.1128/IAI.71.5.2839-2858.2003 12704158 10.1128/IAI.71.5.2839-2858.2003 PMC153285

[25] Plant J, Glynn a a. Locating salmonella resistance gene on mouse chromosome 1. Clin Exp Immunol. 1979;37(1):1–6. 385184 PMC1537656

[26] Johansen F, Pekna M, Natvig Norderhaug I, Haneberg B, Heitala M, Krajci P, et al Absence of Epithelial Immunoglobulin A Transport, with Increased Mucosal Leakiness, in Polymeric Immunoglobulin Receptor / Secretory Component–deficient Mice. J Exp Med. 1999;190(7):915–21. 10510081 10.1084/jem.190.7.915 PMC2195652

[27] Uren TK, Johansen F-E, Wijburg OLC, Koentgen F, Brandtzaeg P, Strugnell R a. Role of the polymeric Ig receptor in mucosal B cell homeostasis. J Immunol. 2003;170(5):2531–9. 12594279 10.4049/jimmunol.170.5.2531

[28] Reikvam DH, Derrien M, Islam R, Erofeev A, Grcic V, Sandvik A, et al Epithelial-microbial crosstalk in polymeric Ig receptor deficient mice. Eur J Immunol. 2012;42(11):2959–70. doi: 10.1002/eji.201242543 22865203 10.1002/eji.201242543

[29] Palm NW, de Zoete MR, Cullen TW, Barry NA, Stefanowski J, Hao L, et al Immunoglobulin A Coating Identifies Colitogenic Bacteria in Inflammatory Bowel Disease. Cell. 2014;158(5):1000–10. doi: 10.1016/j.cell.2014.08.006 25171403 10.1016/j.cell.2014.08.006 PMC4174347

[30] Clarke LL. A guide to Ussing chamber studies of mouse intestine. Am J Physiol Gastrointest Liver Physiol. 2009;296(6):G1151–66c. doi: 10.1152/ajpgi.90649.2008 19342508 10.1152/ajpgi.90649.2008 PMC2697950

[31] Kato-Nagaoka N, Shimada S, Yamakawa Y, Tsujibe S, Naito T, Setoyama H, et al Enhanced differentiation of intraepithelial lymphocytes in the intestine of polymeric Ig receptor-deficient mice. Immunology. 2015;146(1):59–69. doi: 10.1111/imm.12480 25967857 10.1111/imm.12480 PMC4552501

[32] Endt K, Stecher B, Chaffron S, Slack E, Tchitchek N, Benecke A, et al The microbiota mediates pathogen clearance from the gut lumen after non-typhoidal Salmonella diarrhea. PLoS Pathog. 2010;6(9):e1001097 doi: 10.1371/journal.ppat.1001097 20844578 10.1371/journal.ppat.1001097 PMC2936549

[33] Maw J, Meynell GG. The true division and death rates of Salmonella Typhimurium in the mouse spleen determined with superinfecting phage P22. Br J Exp Pathol. 1968;49(6):597–613. 4884387 PMC2093912

[34] Hormaeche CE. The in vivo division and death rates of Salmonella Typhimurium in the spleens of naturally resistant and susceptible mice measured by the superinfecting phage technique of Meynell. Immunology. 1980;41(4):973–9. 7007218 PMC1458291

[35] Mittrucker HW, Raupach B, Kohler a, Kaufmann SH. Cutting edge: role of B lymphocytes in protective immunity against Salmonella Typhimurium infection. J Immunol. 2000;164(4):1648–52. 10657605 10.4049/jimmunol.164.4.1648

[36] de Jong HK, Parry CM, van der Poll T, Wiersinga WJ. Host-Pathogen Interaction in Invasive Salmonellosis. PLoS Pathog. 2012;8(10):1–9. 10.1371/journal.ppat.1002933 PMC3464234 23055923

[37] Tam MA, Rydström A, Sundquist M, Wick MJ. Early cellular responses to Salmonella infection: Dendritic cells, monocytes, and more. Immunol Rev. 2008;225(1):140–62. 18837781 10.1111/j.1600-065X.2008.00679.x

[38] Loomis WP, Johnson ML, Brasfield A, Blanc MP, Yi J, Miller SI, et al Temporal and anatomical host resistance to chronic Salmonella infection is quantitatively dictated by nramp1 and influenced by host genetic background. PLoS One. 2014;9(10). 10.1371/journal.pone.0111763 PMC4211889 25350459

[39] Borrego A, Peters LC, Jensen JR, Ribeiro OG, Koury Cabrera WH, Starobinas N, et al Genetic determinants of acute inflammation regulate Salmonella infection and modulate Slc11a1 gene (formerly Nramp1) effects in selected mouse lines. Microbes Infect. 2006;8(12–13):2766–71. doi: 10.1016/j.micinf.2006.08.005 17035062 10.1016/j.micinf.2006.08.005

