# Supplementary figures and images for: Enhanced survival following oral and systemic Salmonella enterica serovar Typhimurium infection in polymeric immunoglobulin receptor knockout mice
Source: PLoS One. 2018 Jun 1;13(6):e0198434. doi: 10.1371/journal.pone.0198434 (PMC5983570; doi:10.1371/journal.pone.0198434)

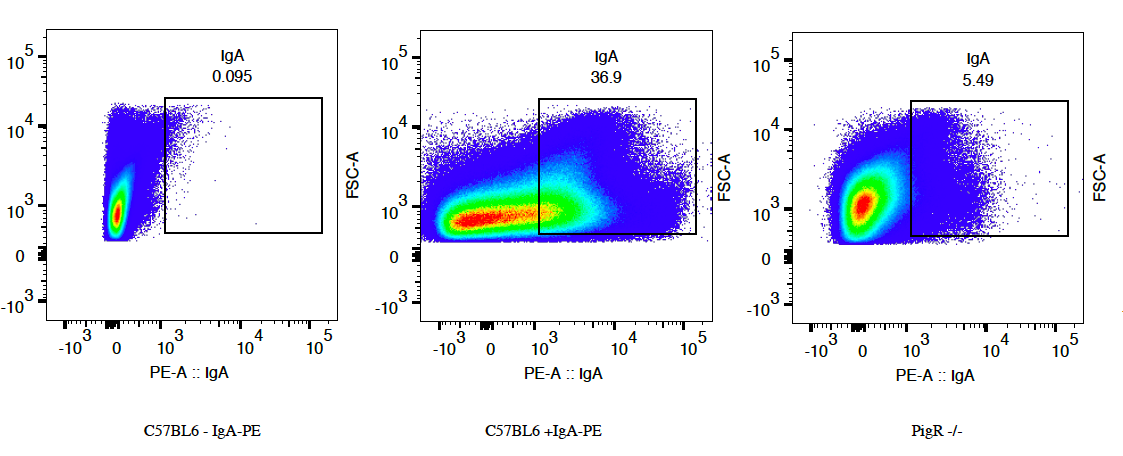

Supplement: S1 Fig — (A) Isotype control staining of C57BL6 stool. (B) Representative plot of IgA staining of bacteria in stool of C57BL6 mouse. (C) Representative plot of IgA staining of bacteria in stool of pIgR KO mouse. (TIFF) [file pone.0198434.s001.tiff]

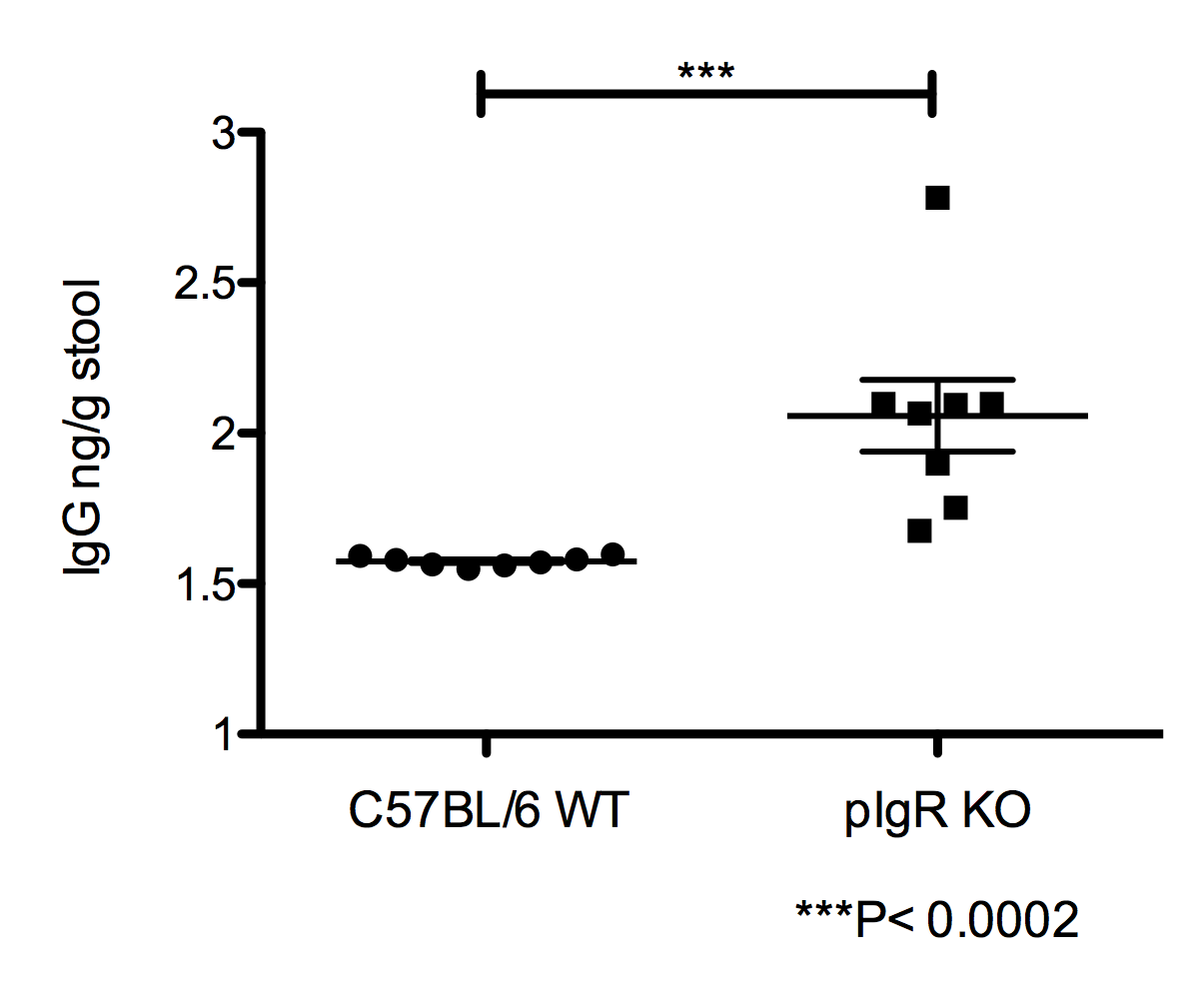

Supplement: S2 Fig — Stool IgG is significantly increased in knockout mice compared to control mice (P<0.0002, Mann-Whitney). Data from eight pIgR KO mice and age-matched WT controls. (TIFF) [file pone.0198434.s002.tiff]

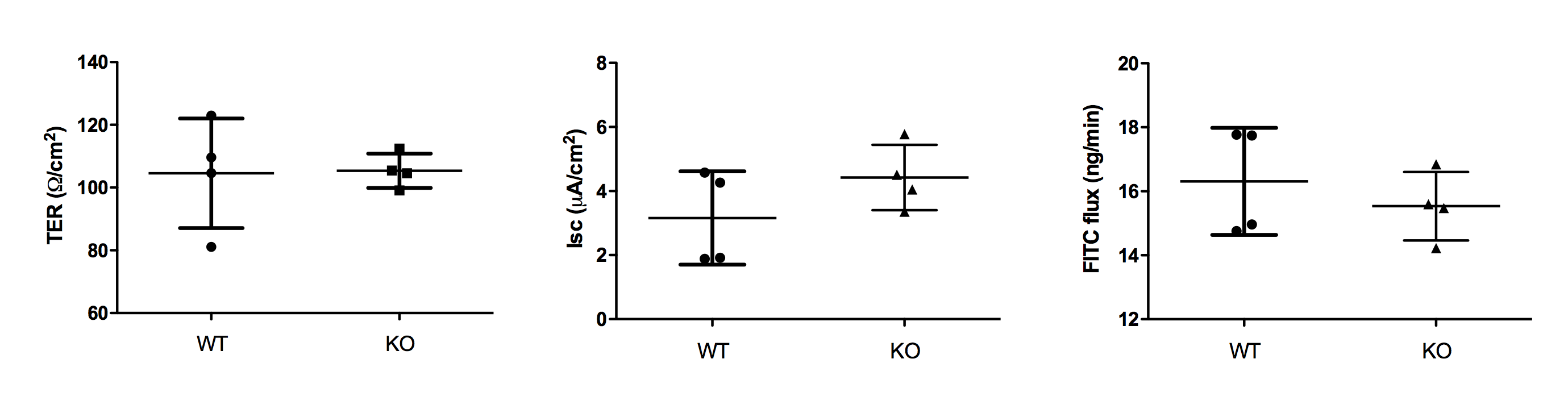

Supplement: S3 Fig — Ussing chamber studies of colonic sections revealed no differences in (A) transepithelial resistance, (B) short circuit current, or (C) FITC-dextran flux. (TIFF) [file pone.0198434.s003.tiff]

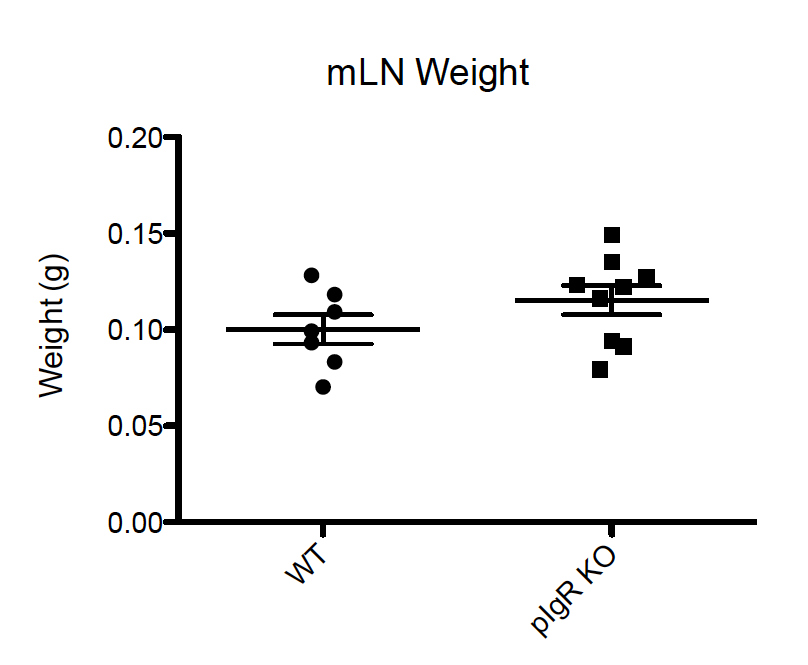

Supplement: S4 Fig — No difference was detected in the weight of mesenteric lymph nodes of pIgR KO mice compared to WT mice. (TIFF) [file pone.0198434.s004.tiff]

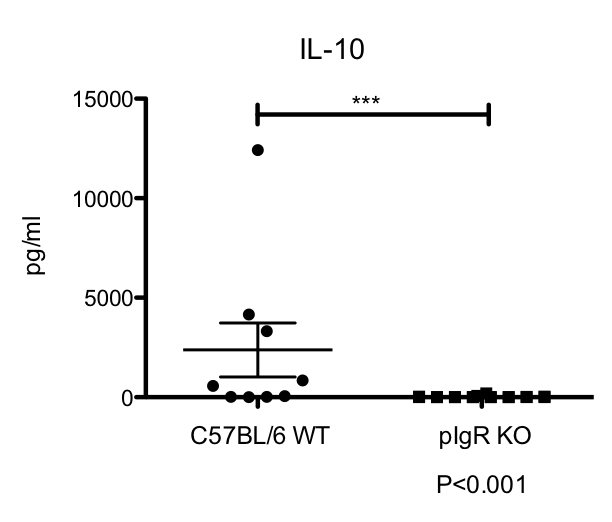

Supplement: S5 Fig — (TIFF) [file pone.0198434.s005.tiff]

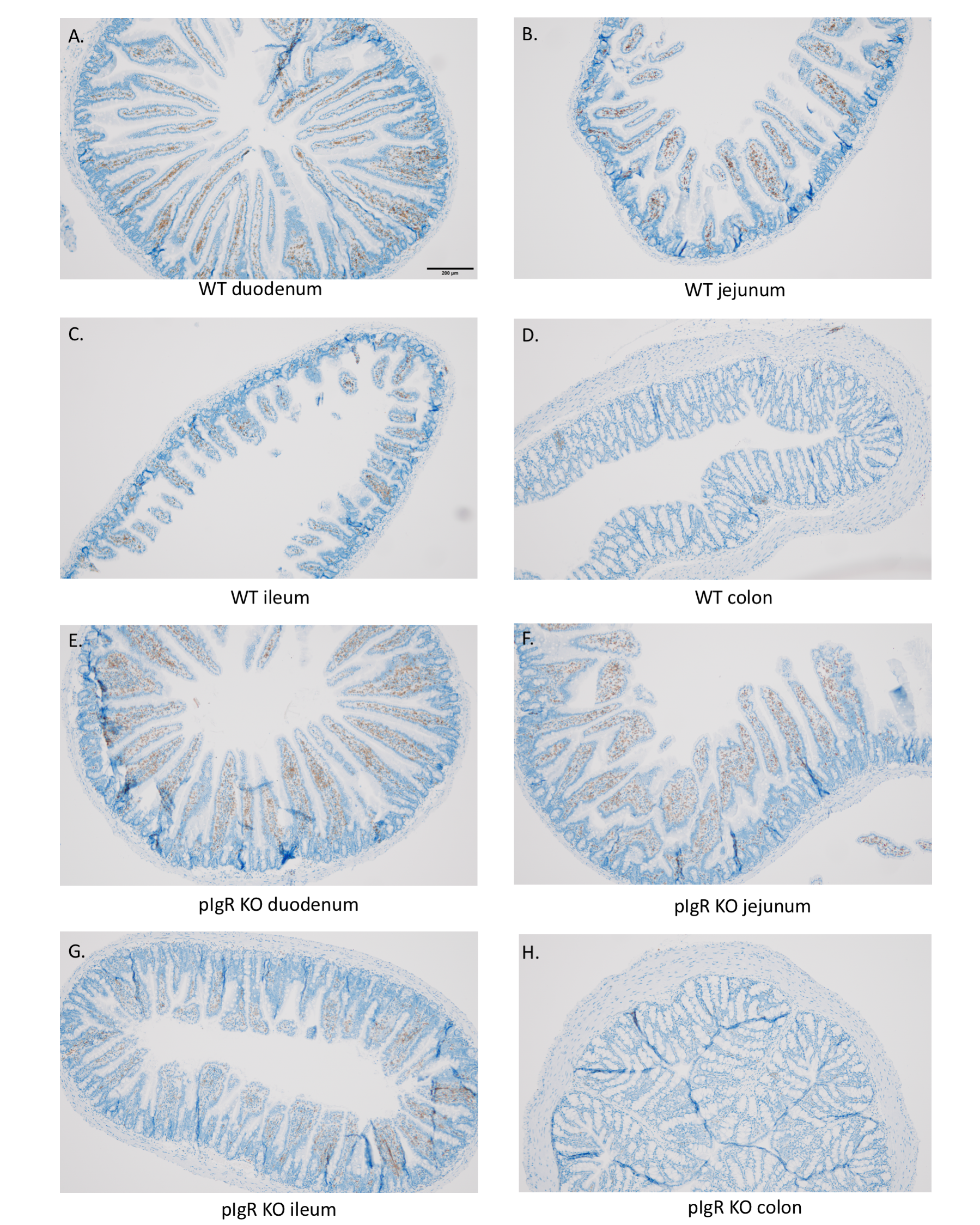

Supplement: S6 Fig — (A) duodenum, (B) jejunum, (C) ileum, (D) colon of wildtype mice and (E) duodenum, (F) jejunum, (G) ileum, (H) colon of pIgR KO mice. Representative images chosen from eight pIgR KO mice and age-matched WT controls. (TIFF) [file pone.0198434.s006.tiff]

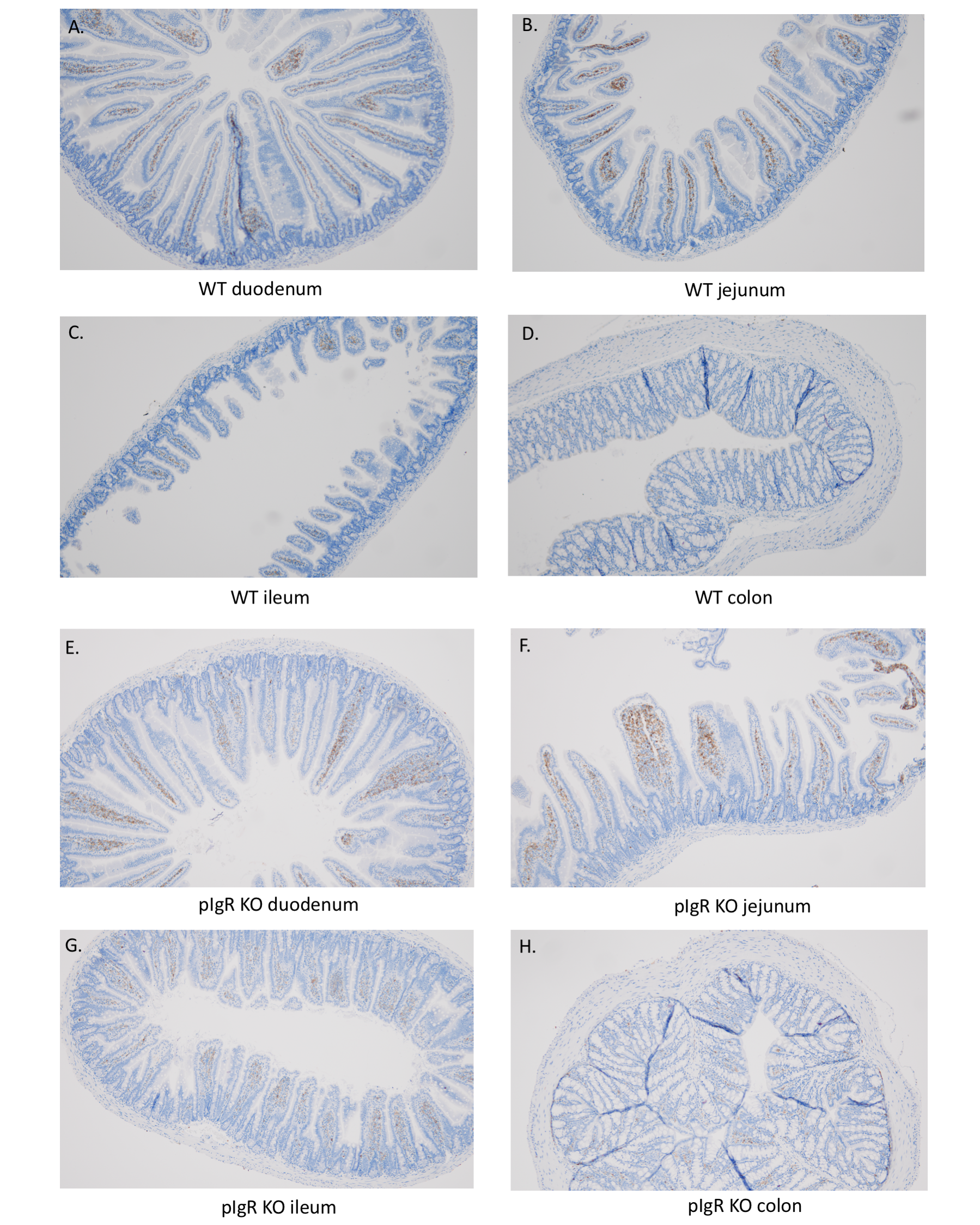

Supplement: S7 Fig — (A) duodenum, (B) jejunum, (C) ileum, (D) colon of wildtype mice and (E) duodenum, (F) jejunum, (G) ileum, (H) colon of pIgR KO mice. Representative images chosen from eight pIgR KO mice and age-matched WT controls. (TIFF) [file pone.0198434.s007.tiff]

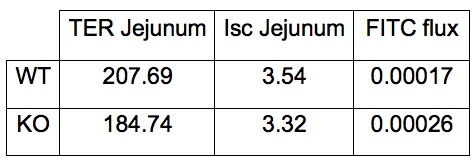

Supplement: S1 Table — (TIFF) [file pone.0198434.s008.tiff]
